# Publisher Correction: High-temperature concomitant metal-insulator and spin-reorientation transitions in a compressed nodal-line ferrimagnet Mn_3_Si_2_Te_6_

**DOI:** 10.1038/s41467-024-49413-8

**Published:** 2024-06-20

**Authors:** Resta A. Susilo, Chang Il Kwon, Yoonhan Lee, Nilesh P. Salke, Chandan De, Junho Seo, Beomtak Kang, Russell J. Hemley, Philip Dalladay-Simpson, Zifan Wang, Duck Young Kim, Kyoo Kim, Sang-Wook Cheong, Han Woong Yeom, Kee Hoon Kim, Jun Sung Kim

**Affiliations:** 1 Department of Physics, Pohang University of Science and Technology, Pohang, Korea; 2 Center for Artificial Low Dimensional Electronic Systems, Institute for Basic Science (IBS), Pohang, Korea; 3 Department of Physics and Astronomy, CeNSCMR, Seoul National University, Seoul, Korea; 4 Departments of Physics, University of Illinois Chicago, Chicago, IL USA; 5 Departments of Chemistry, University of Illinois Chicago, Chicago, IL USA; 6 Department of Earth and Environmental Sciences, University of Illinois Chicago, Chicago, IL USA; 7 Center for High Pressure Science and Technology Advanced Research, Shanghai, China; 8 Korea Atomic Energy Research Institute (KAERI), Daejeon, Korea; 9 Laboratory of Pohang Emergent Materials, Pohang Accelerator Laboratory, Pohang, Korea; 10 Rutgers Center for emergent Materials and Department of Physics and Astronomy, Rutgers University, New Brunswick, NJ USA

**Keywords:** Magnetic properties and materials, Electronic properties and materials

Correction to: *Nature Communications* 10.1038/s41467-024-48432-9, published online 11 May 2024

In the original version of this article Kee Hoon Kim was not listed as a corresponding author. The article has been corrected.

