# Supplementary material for: High-temperature concomitant metal-insulator and spin-reorientation transitions in a compressed nodal-line ferrimagnet Mn3Si2Te6
Source: Nat Commun. 2024 May 11;15:3998. doi: 10.1038/s41467-024-48432-9 (PMC11088669; doi:10.1038/s41467-024-48432-9)
Supplement: Supplementary file 1 — Supplementary Information [file 41467_2024_48432_MOESM1_ESM.pdf]

Supplementary Information: High-temperature concomitant metal-insulator and spin-reorientation transitions in a compressed nodal-line ferrimagnet $Mn_3Si_2Te_6$

Resta A. Susilo,[1,*] Chang Il Kwon,[1,2,*] Yoonhan Lee,[3,*] Nilesh P. Salke,[4] Chandan De,[2] Junho Seo,[1,2] Beomtak Kang,[1,2] Russell J. Hemley,[4,5,6] Philip Dalladay-Simpson,[7] Zifan Wang,[7] Duck Young Kim,[7] Kyoo Kim,[8] Sang-Wook Cheong,[9,10] Han Woong Yeom,[1,2] Kee Hoon Kim,[3,†] and Jun Sung Kim[1,2,‡]

[1]*Department of Physics, Pohang University of Science and Technology, Pohang, Korea*

[2]*Center for Artificial Low Dimensional Electronic Systems, Institute for Basic Science (IBS), Pohang, Korea*

[3]*Department of Physics and Astronomy, CeNSCMR, Seoul National University, Seoul, Korea*

[4]*Departments of Physics, University of Illinois Chicago, Chicago, USA*

[5]*Departments of Chemistry, University of Illinois Chicago, Chicago, USA*

[6]*Department of Earth and Environmental Sciences, University of Illinois Chicago, Chicago, USA*

[7]*Center for High Pressure Science and Technology Advanced Research, Shanghai, China*

[8]*Korea Atomic Energy Research Institute (KAERI), Daejeon, Korea*

[9]*Laboratory of Pohang Emergent Materials, Pohang Accelerator Laboratory, Pohang, Korea*

[10]*Rutgers Center for emergent Materials and Department of Physics and Astronomy, Rutgers University, NJ, USA*

Supplementary Note 1. Transport properties of $Mn_3Si_2Te_6$ at high pressures

The metal-insulator transition of $Mn_3Si_2Te_6$ at high pressures without external magnetic fields was studied by the activation behaviors of the temperature-dependent resistivity, as shown in Fig. 1d of the main text. The systematic suppression of the thermally activated conduction can be understood by the Arrhenius model $\rho_{ab}(T) \sim \exp(\Delta/k_B T)$, where Δ is the activation gap. From fitting the ρ_{ab} data to the model in the temperature range of 70-20 K, we extracted the activation gap as a function of pressure (Fig. S1b). Below $\sim$ 20 K, variable range hopping conduction dominates, as typically found in extrinsic semiconductors and $Mn_3Si_2Te_6$ at ambient pressure [1]. Consistent with the decrease in the overall resistivity at 20 K, the activation gap Δ falls continuously with pressure and closes near $P_c \sim$ 14 GPa, indicating the pressure-driven metal-insulator transition (MIT).

The temperature-dependent resistivity ρ_{ab} at different pressures and magnetic fields along the c axis ($H \parallel c$) reveals a reduction in magnetoresistivity with incresing pressure, as shown

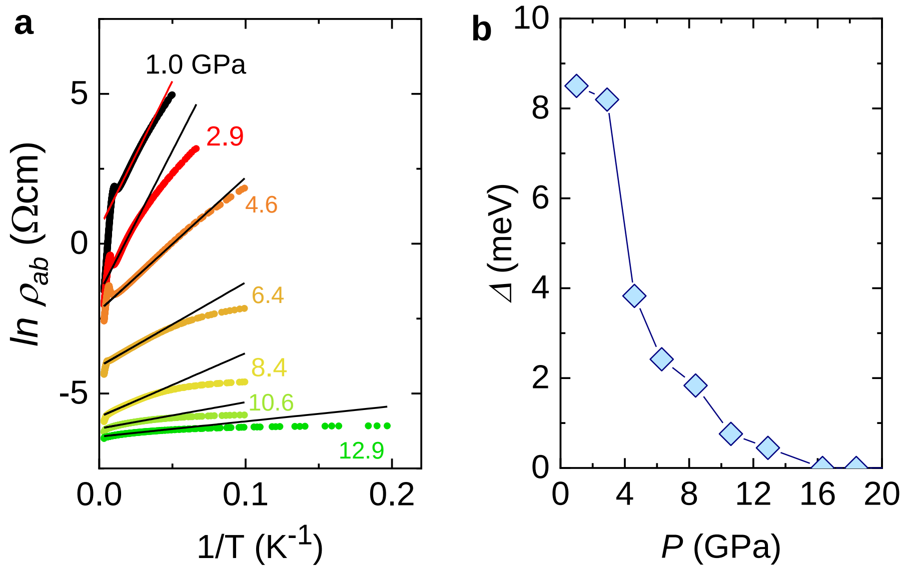

Fig. S1. **Pressure-induced insulator-metal transition in $Mn_3Si_2Te_6$.** (a) Log resistivity as a function of the inverse temperature for $Mn_3Si_2Te_6$ at various pressures. Solid lines are the fitting to the data with the activation model, $\rho(T) \sim \exp(\frac{\Delta}{k_B T})$, where Δ is the activation energy. (b) The pressure-dependence of activation energy of $Mn_3Si_2Te_6$.

* equal contribution

† optopia@snu.ac.kr

‡ js.kim@postech.ac.kr

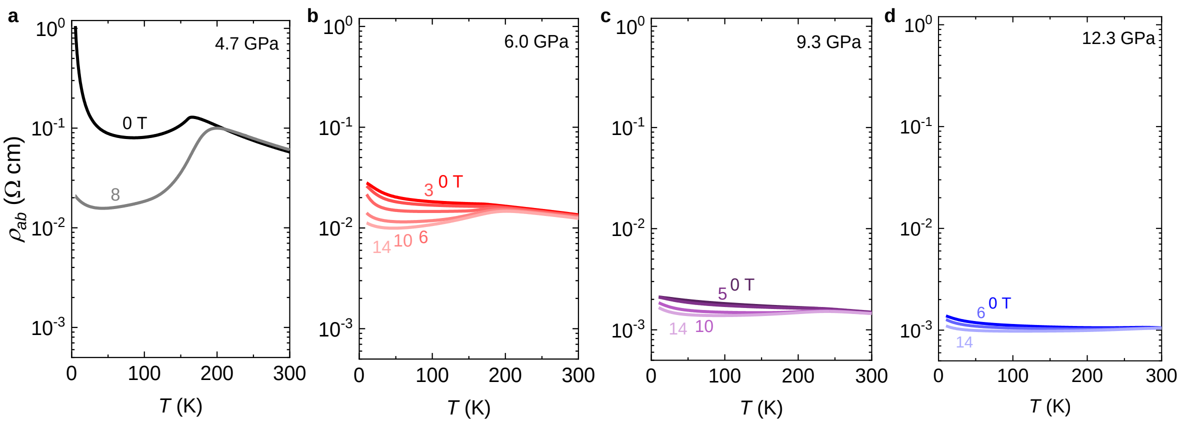

Fig. S2. **Weakening of magnetoresistivity by pressure in $Mn_3Si_2Te_6$.** The temperature dependendent of the in-plane resistivity at different magnetic fields and at (a) 4.7 GPa, (b) 6.0 GPa, (c) 9.3 GPa and (d) 12.3 GPa, with the magnetic fields applied along the c-axis.

in Figs. S2a-d. Due to the colossal angular magnetoresistance (CAMR) effect [1], the resistivity drops by seven orders of magnitude at $T = 2$ K for $H \parallel c \sim 2$ T at ambient pressure. This resistivity drop by $H \parallel c$ is drastically suppressed with pressure. For example, at $P =$ 4.7 GPa, the corresponding resistivity drop by $H \parallel c \sim 8$ T is only two orders of magnitude (Fig. S2a). Just below the critical pressure P_c, $\rho_{ab}(T)$ data at *e.g.* $P = 12.3$ GPa (Fig. S2d) exhibit little variation even with $H = 14$ T. As described in the main text, the substantial reduction in both the resistivity and the magnetoresistance is consistent with the fact that the Te bands with nodal-line band degeneracy approach the Fermi level under pressure.

Supplementary Note 2. Hall response of $Mn_3Si_2Te_6$

Below P_C, the Hall conductivity ($\sigma_{xy}(H) = \rho_{xy}/(\rho_{xx}^2 + \rho_{xy}^2)$) shows a non-linear field dependence which cannot be explained by the conventional Hall response. Moreover, because the out-of-plane magnetization tends to grow linearly with magnetic field, the behaviour of $\sigma_{xy}(H)$ clearly deviates from the conventional anomalous Hall response, proportional to magnetization. In the case of $Mn_3Si_2Te_6$, the field-induced MIT due to the opening of the spin-orbit coupling gap may account for this unusual Hall response.

Using the single carrier model, the Hall conductivity is described by $\sigma_{xy} = \mu^2 neH/(1 + (\mu H)^2)$, where μ and n are the carrier mobility and density, respectively. For $H \parallel c$ the spin

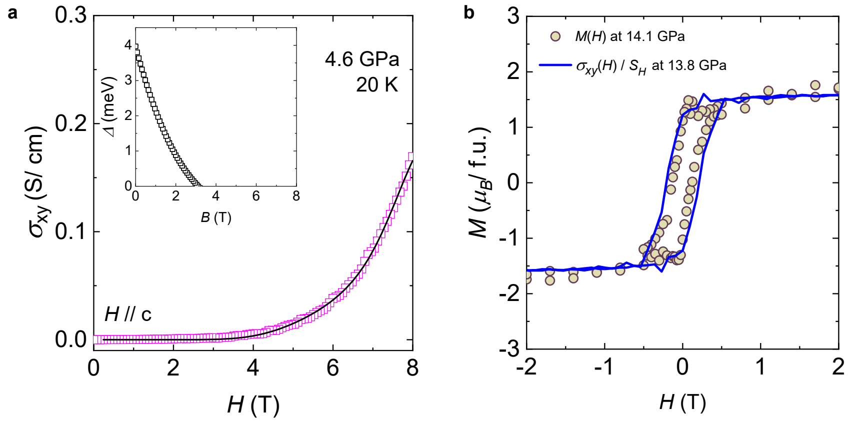

Fig. S3. **Hall response of $Mn_3Si_2Te_6$.** (a) Hall conductivity of $Mn_3Si_2Te_6$ at 20 K and 4.6 GPa (below P_C). Solid line represents a fit to the data using the model described in the text. Inset shows the derived activation gap as a function of magnetic field. (b) Hall conductivity at 10 K and ∼14 GPa (below P_C). The field-dependent magnetization $M(H)$ is nicely reproduced by $\sigma_{xy}(H)$ with a scaling factor S_H following a relation $\sigma_{xy}(H) = S_H M(H)$

gradually rotates from the ab-plane towards the c-axis, and the activation gap of $Mn_3Si_2Te_6$ exhibits a strong magnetic field dependent $\Delta(H)$. In this case, the carrier density and mobility are no longer constant but will instead be strongly dependent on the magnetic field. Here, we describe the field dependence μ and n according to the Arrhenius equation *i.e.* $\mu(H) \approx \mu_0 \exp(-\frac{\Delta(H)}{k_B T})$ and $n(H) \approx n_0 \exp(-\frac{\Delta(H)}{k_B T})$, where $\Delta(H)$ is assumed to follow a third-order polynomial function *e.g.* $\Delta(H) = \Delta_0 - aH - bH^2 - cH^3$ with a, b, c are constants.

Employing this model, we can reproduce the $\sigma_{xy}(H)$ data at 4.6 GPa as shown in Fig. S3(a). Here, Δ_0 is set to be ∼4 meV, based on the $\rho(T)$ data, and n_0 is taken from the recent work by Ni *et al.* [2], which is on the order of $\sim 10^{16}$ cm^{-3} at ambient pressure. The extracted activation gap $\Delta(H)$ closes above 3 T (the inset of Fig.S3(a)), consistent with the magnetic field-induced MIT. These results reveal that the seemingly unusual Hall response below P_C can be well explained by the thermally-excited carriers across the strongly field-dependent activation gap $\Delta(H)$.

As discussed in the main text, the resistivity anomaly disappears above P_c in the metallic state, therefore a different criterion was used to determine T_C. Once the system enters the metallic state above P_c, the field dependence Hall resistivity $\rho_{xy}(H)$ shows a clear square-shaped behavior indicating the perpendicular magnetic anisotropy above P_c. Since

ρ_{xy} is dominated by the anomalous contribution, i.e., $\rho_{xy} \simeq \rho_{xy}^A$, the corresponding Hall conductivity, $\sigma_{xy}(H)$, can be scaled nicely with $M(H)$ as shown in Fig. S3(b). Assuming that the scaling factor $S_H = \sigma_{xy}(H)/M(H)$ is almost temperature-independent, the net magnetization $M(H)$ can therefore be represented by $\sigma_{xy}(H)$ and thus in principle, T_c can be determined from the temperature dependence of $\sigma_{xy}(H)$. This approach enabled us to determine T_c above P_c leading to a complete magnetic phase diagram presented the main text.

Supplementary Note 3. Magnetic properties of $Mn_3Si_2Te_6$ below the critical pressure

Figure S4 presents magnetic susceptibility and magnetization at various pressures measured with the applied magnetic fields for $H \parallel ab$ and $H \parallel c$. In good agreement with the

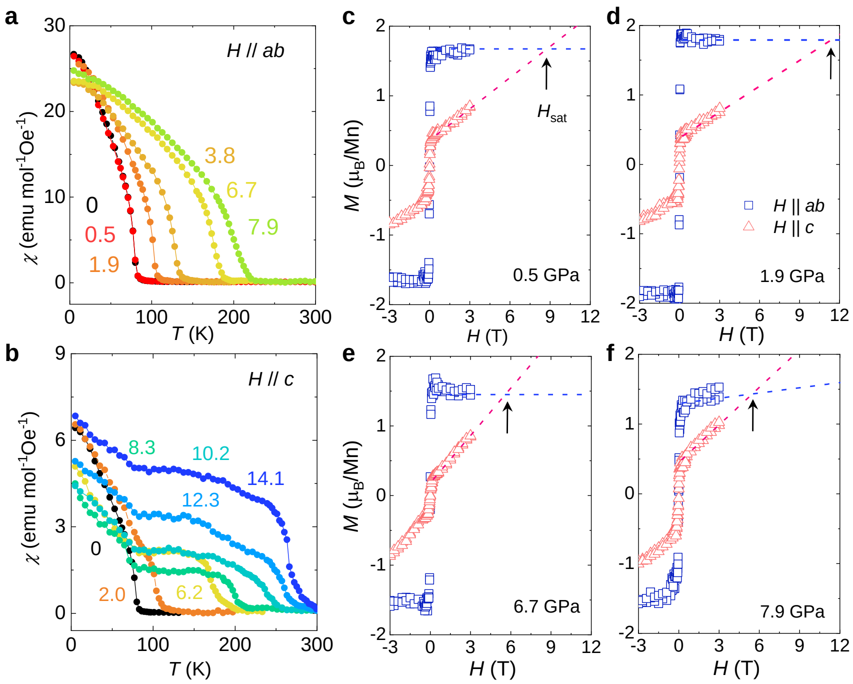

Fig. S4. **Magnetic properties of $Mn_3Si_2Te_6$ at various pressures.** (a,b) Magnetic susceptibility measured with the applied parallel (a) and perpendicular (b) to the *ab*-plane. (c-f) Isothermal magnetization curves at 5 K for both $H \parallel c$ and $H \parallel ab$ at (d) 0.5 GPa, (e) 1.9 GPa, (f) 6.7 GPa and (g) 7.9 GPa. Dashed lines are extrapolated magnetization curves to high magnetic fields. The estimated saturation fields H_{sat} is indicated by the arrows.

resistivity data discussed in the main text, the paramagnetic-ferrimagnetic transition (T_c) shifts towards high temperature with increasing pressure, approaching room temperature at $P = 14.1$ GPa close to the critical pressure $\sim P_c$ for the MIT (Figs. S4 a-b).

The pressure-dependent change in the magnetic anisotropy is observed in the isothermal magnetization for both $H \parallel c$ and $H \parallel ab$ at various pressures up to 8 GPa (Figs. S4 c-f). Due to the limitation of the applied magnetic field up to 3 T, the saturation field H_{sat} for $H \parallel c$ is estimated by the intersection point of both $M(H)$ curves extrapolated to higher fields, as indicated by the arrows in Figs. S4 c-f. The obtained $H_{sat} \sim 9$ T at 0.5 GPa is consistent with $H_{sat} \sim 10$ T measured at ambient pressure [1, 2]. Upon increasing pressure, H_{sat} decreases down to $H_{sat} \sim 5$ T at around 8 GPa, while the saturation magnetic moment M_{sat} is also somewhat reduced from 1.6 μ_B to 1.4 μ_B. Accordingly, the magnetic anisotropy energy calculated from the relation $H_{sat} = 2K/Msat$ decreases with pressure from $\sim$0.7 J cm^{-3} at ambient pressure to $\sim$0.4 J cm^{-3} at 7.9 GPa. These findings clearly indicate that in-plane magnetic anisotropy is suppressed with pressure, consistent with spin-reorientation transition at the critical pressure P_c.

Supplementary Note 4. Raman spectra of $Mn_3Si_2Te_6$ under pressure

In order to complement the pressure-dependent XRD results, we investigate the vibrational properties of $Mn_3Si_2Te_6$ under pressure via Raman spectroscopy in a diamond anvil cell. Raman spectra were obtained through a home-built Raman spectroscopy setup comprising a 750-mm monochromator and a liquid-nitrogen-cooled CCD (Princeton Instruments). A 633-nm He–Ne laser was utilized as the excitation source. Silicon oil was used as a pressure transmitting medium and ruby balls as pressure determinants [3].

The collected Raman spectra at room temperature are displayed in Fig. S5a as a contour plot. The Raman spectra of $Mn_3Si_2Te_6$ are dominated by five strongest Raman modes in the frequency range below 250 cm^{-1} *e.g.* at $\sim$59, 66, 96, 108 and 153 cm^{-1} which have been identified as the $E_{\mathrm{g}}^2, A_{1\mathrm{g}}^1, E_{\mathrm{g}}^5, A_{1\mathrm{g}}^2$ and $A_{1\mathrm{g}}^4$ modes, respectively [4]. Upon increasing pressure, three Raman modes, the $A_{1\mathrm{g}}^1, A_{1\mathrm{g}}^2$ and $A_{1\mathrm{g}}^4$ modes, shift towards higher frequency, while the E_{g}^2 and E_{g}^5 modes exhibit a nearly constant dependence. This observation is consistent with the anisotropic lattice response seen from XRD data, in which the c-axis is more compressible than the in-plane direction. Therefore, the $A_{1\mathrm{g}}^1, A_{1\mathrm{g}}^2$ and $A_{1\mathrm{g}}^4$ modes can be assigned to the out-of-plane vibration, whereas the E_{g}^2 and E_{g}^5 modes correspond to the

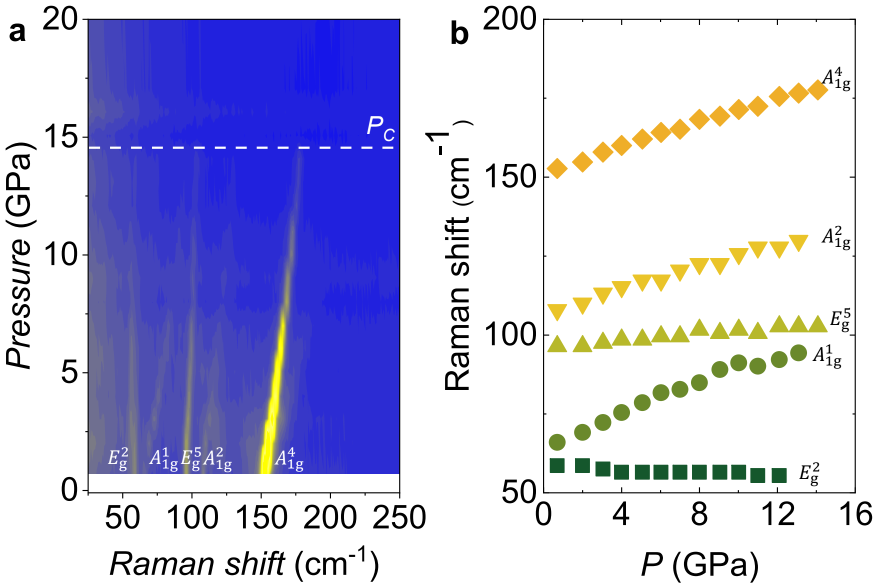

Fig. S5. **Raman spectra of $Mn_3Si_2Te_6$ at high pressure.** (a) Contour plot of Raman spectra collected at various pressures up to 20 GPa. (b) Pressure-dependence frequencies of five dominant Raman modes.

in-plane vibration. Above the critical pressure $P_c \sim 14$ GPa, all Raman peaks disappear due to a significant reduction of laser penetration depth as the conductivity increases in the metallic state.

Supplementary Note 5. High pressure structure of $Mn_3Si_2Te_6$ above P_c

To deduce the high pressure phase, first, the lattice parameters and crystal symmetry were indexed and determined using the DICVOL14 software [5] and the FOX software [6]. This procedure results in several candidate structures *i.e.* hexagonal $P6_3/mmc$, orthorhombic $Cmcm$, monoclinic $C2/m$ and $C2/c$ structures as shown in Figs. S6b-e. Other monoclinic cells with anomalously small unit cell volume and the original trigonal $P\bar{3}1c$ structure with doubled-unit cell along the c-axis were also found but excluded. The four possible candidates structure were then used to fit the X-ray diffraction patterns above P_c. These structural candidates reproduce the diffraction patterns quite well, although the fitting result with the monoclinic $C2/c$ structure is superior, compared to other structures (Table S1). The fitting results using the monoclinic $C2/c$ structure is shown in Fig. S6a.

We then utillized density-functional-theory (DFT) calculations to estimate the relative

enthaply energies of these candidate structures, as summarized in Table S1. We relaxed the unit cell shape for the candidate structures ($P6_3/mmc$, $CmCm$, $C2/m$, $C2/c$) and the low pressure trigonal structure ($P\bar{3}1c$), while keeping the experimentally-determined unit cell volume. For total energy evaluation, we used the k point mesh of 12×6×6 and energy cutoff of 450 eV. We found that the candidate hexagonal structure ($P6_3/mmc$) structure is strongly unstable and the total energy cannot be defined. For the $CmCm$ and $C2/m$ structures, full structural relaxation leads to a local distortion which tends to break the $CmCm$ and $C2/m$ symmetries. Therefore, we only relaxed the out-of plane of coordinates of each atoms for these cases. The resulting total energies are found to be too high as compared to that of the low pressure structure ($P\bar{3}1c$). Because both the hexagonal and orthorhombic structures have much higher energies than the low-pressure structure, these structures are unlikely to be viable candidates for the high-pressure phase. In fact, the structural transformation to a hexagonal or an orthorhombic structure involves a significant displacement of Te atom in the ab-plane, modifying the local $MnTe_6$ structure from octahedra to a more rigid trigonal

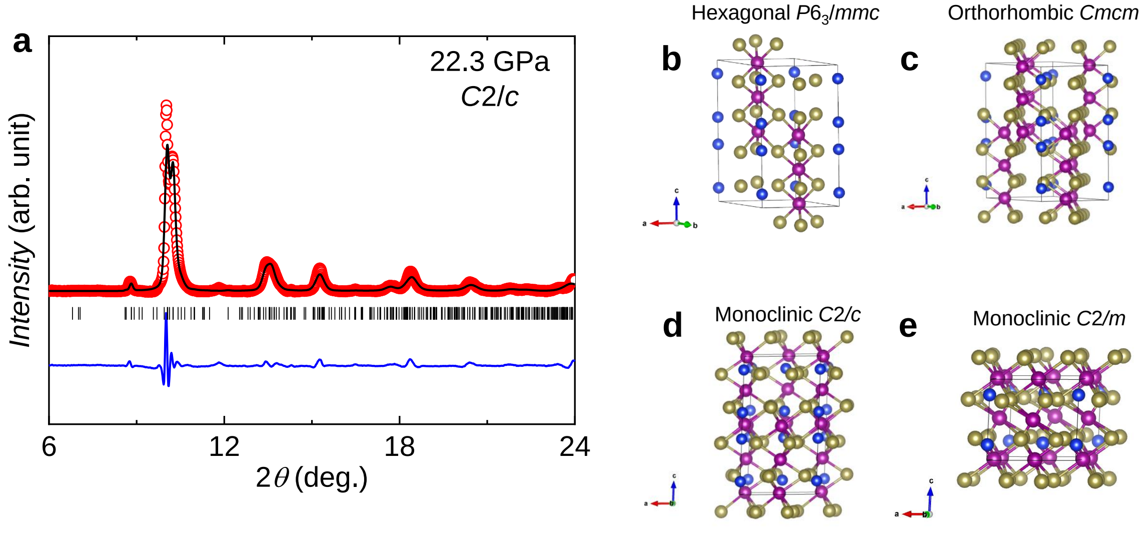

Fig. S6. **Le Bail fitting to the X-ray diffraction pattern above P_C with a possible high pressure structural candidate.** (a) Le Bail fitting to the X-ray diffraction pattern taken at 22.3 GPa using the monoclinic $C2/c$ space group. Possible candidates for high pressure crystal structure of $Mn_3Si_2Te_6$ above P_C: (b) hexagonal $P6_3/mmc$, (c) orthorhombic $Cmcm$, (d) monoclinic $C2/c$ and (e) $C2/m$ structures.

Table. S1. **Comparison of Le Bail fitting results to the diffraction pattern at 22.3 GPa**. All structural models provide good fits to the observed pattern, but the monoclinic $C2/c$ space group has the lowest χ^2 and R-factors. Total energies per formula unit of the candidate structures relative to that of low-pressure trigonal structure are listed together with the calculated magnetization M.

Space group	a (Å)	b (Å)	c (Å)	β (°)	χ^2	R_p	R_{wp}	E-E_0 (eV/f.u.)	M (μB/f.u.)
$P6_3/mmc$	6.2547(8)	6.2547(8)	11.779(6)	90	0.71	23.8	20.8	–	–
$Cmcm$	6.417(1)	10.813(2)	11.563(6)	90	0.59	21.5	19.2	9.671	3.82
$C2/m$	6.427(1)	10.814(2)	5.803(3)	92.7(1)	0.67	23.1	20.4	6.512	4.25
$C2/c$	6.438(1)	10.816(2)	11.625(5)	94.1(1)	0.46	19.5	17.0	-0.044	3.99

prism [7], which is unfavorable at high pressure. In contrast, we found that the monoclinic $C2/c$ is stable with full relaxation and has the lowest energy than the other candidate structures. This is also consistent with the fact that the monoclinic $C2/c$ structure model yields the best-fitting result of the diffraction pattern. Therefore we conclude that above P_c, $Mn_3Si_2Te_6$ transforms from a trigonal $P\bar{3}1c$ to a monoclinic $C2/c$ structure.

Figure S7 depicts a comparison between these two structures. The monoclinic $C2/c$ structure can be seen as a distorted trigonal $P\bar{3}1c$ structure, in which a single Te site is split into three distinct sites and also the $MnSiTe_3$ layers slide slightly with respect to each other, thereby introducing a monoclinic distortion. Despite the reduction in symmetry from trigonal to monoclinic ones, the overall structural motifs of the low and high pressure phases are identical. In particular, the high pressure structure still maintains the alternating stack of the $MnSiTe_3$ layers and Mn triangular lattice.

In Fig. S8, we present the pressure-dependent lattice parameters of the monoclinic phase above P_c. All the lattice parameters decrease systematically with pressure while the β angle increases with pressure. By normalizing the lattice parameters to those at P_c (Fig. S8b), we found that their relative changes with pressure are nearly the same, indicating isotropic lattice compression in the monoclinic phase above P_c. To directly compare the pressure-dependence of the lattice parameters for both low-pressure trigonal and high-pressure mon-

oclinic phases, we transformed the monoclinic lattice parameters using the pseudo-trigonal system (see Figs. S7b and d). Because the monoclinic $C2/c$ space group is one of the subgroup of the trigonal $P\bar{3}1c$, the monoclinic lattice vectors, $\mathbf{a_m}, \mathbf{b_m}$ and $\mathbf{c_m}$, are related to the trigonal lattice vectors, $\mathbf{a_t}, \mathbf{b_t}$ and $\mathbf{c_t}$, *via* [8, 9]:

$$\mathbf{a_m} = -\mathbf{a_t} + \mathbf{b_t}, \mathbf{b_m} = -\mathbf{a_t} - \mathbf{b_t}, \mathbf{c_m} = \mathbf{c_t}. \tag{1}$$

By taking the reverse transformation of Eq. (1), the corresponding pseudo-trigonal lattice vectors, $\mathbf{a'_t}, \mathbf{b'_t}$ and $\mathbf{c'_t}$ can be written as

$$\mathbf{a'_t} = -\frac{1}{2}\mathbf{a_m} - \frac{1}{2}\mathbf{b_m}, \mathbf{b'_t} = \frac{1}{2}\mathbf{a_m} - \frac{1}{2}\mathbf{b_m}, \mathbf{c'_t} = \mathbf{c_m}. \tag{2}$$

The resulting pseudo-trigonal lattice parameters above P_c are shown in Fig. 3c in the main text.

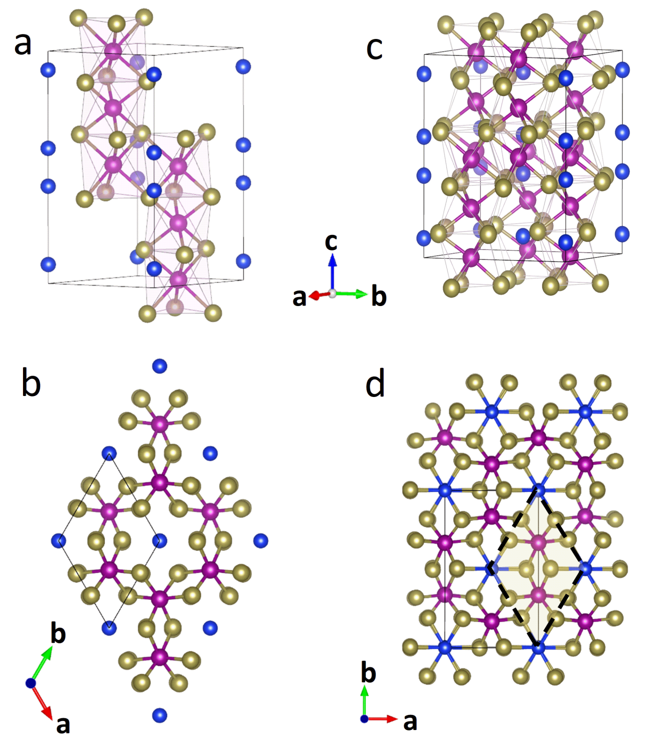

Fig. S7. **Comparison between the low and high pressure phases of $\mathbf{Mn_3Si_2Te_6}$.** (a) The low pressure trigonal $P\bar{3}1c$ structure and (b) its projection onto the *ab*-plane. (c) The high-pressure monoclinic $C2/c$ structure and (d) its projection onto the *ab*-plane. The shaded region in (d) depicts the pseudo-trigonal lattice system as discussed in the text.

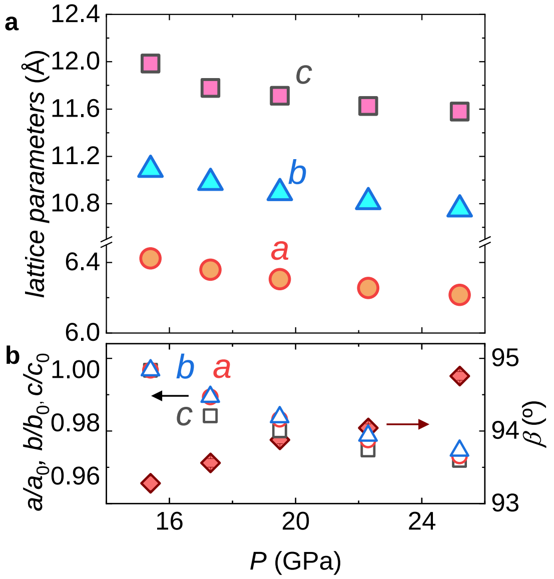

Fig. S8. **Lattice parameters of the high pressure phases of $Mn_3Si_2Te_6$.** (a) The pressure dependences of lattice a, b and c parameters. (b) Normalized lattice parameters and β angle of the monoclinic crystal.

Supplementary Note 6. Variation of bonding distances and angles below P_C

Ferrimagnetism in $Mn_3Si_2Te_6$ results from the competition between three nearest neighbor exchange interactions: (1) interlayer J_1 which connects Mn-Mn of the face-sharing octahedra, (2) intralayer J_2 between Mn-Mn in the edge-sharing octahedra and (3) J_3 that links Mn^{2+} spins in the corner-sharing octahedra (Fig. S9a). Due to a relatively short Mn_1-Mn_2 distance (d_1) of 3.5 Å, the strong AFM direct exchange is expected to dominate the interlayer J_1. For the intralayer J_2, the Mn_1-Te-Mn_1 superexchange will contribute to the overall exchange interaction. Since the Mn_1-Te-Mn_1 bonding angle (θ_2) is close to 90°, the Mn_1-Te-Mn_1 superexchange will be FM in nature and comparable to the Mn-Mn direct exchange. Despite a longer Mn-Mn distance (d_2 ∼4.1 Å), it appears that the Mn_1-Mn_1 direct exchange still dominates the Mn_1-Te-Mn_1 superexchange, resulting in the AFM exchange coupling of J_2. As for the J_3 coupling, because the Mn_1-Mn_2 distance (d_3) is relatively long (∼5.4 Å), the direct Mn_1-Mn_2 exchange interaction is negligible and thus the Mn_1-Te-Mn_2 superexchange dictates the J_3 exchange coupling. However, the Mn_1-Te-Mn_2 bonding angle deviates significantly from 180° ($\theta_3 \sim 137°$), which leads to the AFM superexchange interaction moderate. As a consequence, the hierarchy of three exchange interactions is described by $J_2 \lesssim J_3 \ll J_1$, and the competition between J_2 and J_3 is critical to determine T_c.

The pressure-dependent variations in the Mn-Mn bonding distances (d_1, d_2, and d_3) and Mn-Te-Mn angles (θ_1, θ_2, and θ_3) are presented in Figs. S10b-e. At higher pressures, the Mn_1-Mn_2 vertical distance d_1 decreases faster than the other distances, thereby enhancing the dominant interlayer antiferromagnetic (AFM) interaction J_1. Simultaneously, the horizontal Mn_1-Mn_1 distance d_2 appears to decrease more rapidly than the diagonal Mn_1-Mn_2 distance d_3, resulting in enhanced direct AFM Mn_1-Mn_1 exchange. In addition, the deviation of the Mn_1-Te-Mn_1 angle θ_2 from 90° (Fig. S9d) suppresses the ferromagnetic (FM) Mn_1-Te-Mn_1 superexchange, further enhancing the interlayer AFM exchange coupling J_2. Interestingly, the Mn_1-Te-Mn_2 bonding angle θ_3, which determines the AFM exchange J_3, increases toward 180° due to the strong anisotropic lattice response with pressure. This effect leads to a rapid enhancement of the AFM Mn_1-Te-Mn_2 superexchange interaction and hence J_3. Consequently, the increase in J_3 is dominant over that of J_2 with pressure, resulting in a significant enhancement of T_c in $Mn_3Si_2Te_6$.

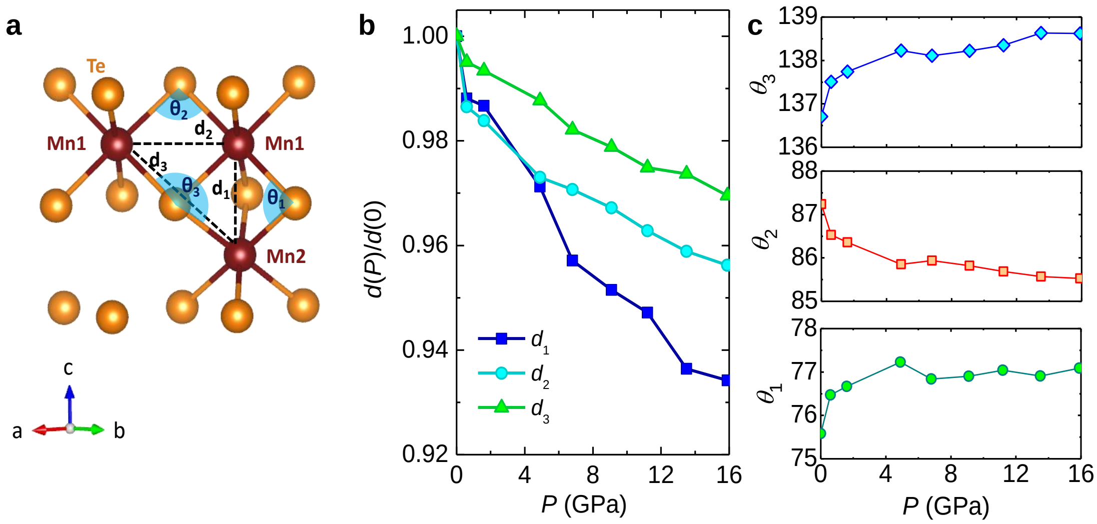

Fig. S9. **Variation of bonding distances and angles of the $MnTe_6$ octahedra with pressure below P_c.** (a) Three $MnTe_6$ octahedra in $Mn_3Si_2Te_6$. Mn-Mn bonding distances associated with the direct exchange are indicated by d_1, d_2, d_3, whereas the Mn-Te-Mn bonding angles relevant for the Mn-Te-Mn superexchange paths are labelled by $\theta_1, \theta_2, \theta_3$. (b) Pressure-dependence of the normalized Mn-Mn bonding distances. (c) Pressure-dependence of the Mn-Te-Mn bonding angles contributed to the Mn-Te-Mn superexchange paths.

Supplementary Note 7. High pressure effects on Se-doped $Mn_3Si_2(Te_{1-x}Se_x)_6$

According to the previous studies [1] Se-doping of $Mn_3Si_2(Te_{1-x}Se_x)_6$ ($x = 0.2$) exhibits a larger activation gap and lower $T_c \sim 68$ K compared to the undoped ($T_c \sim 78$ K). The deeper impurity band position relative to the Fermi level likely contributes to the more insulating nature in Se-doped $Mn_3Si_2Te_6$. These results are consistent with the fact that full replacement of Te by Se in $Mn_3Si_2Se_6$ leads to more insulating behavior as shown in the previous study [10]. Moreover, Se-doping reduces the a and c lattice parameters by $\sim 1\%$ and $\sim 3.5\%$, respectively, due to the smaller atomic size of Se than Te. While the decreases of the c-axis parameter enhances the interlayer AFM exchange J_1, it also reduces the relative difference between the intralayer J_2 and J_3 exchanges, possibly as a result of changes in the Mn-Te(Se) bond angle and distance. This leads to a greater frustrated magnetic exchange that reduces T_c.

At high pressures up to ∼24 GPa, the strong insulating behavior of the Se-doped $Mn_3Si_2(Te_{1-x}Se_x)_6$ ($x = 0.2$) is systematically suppressed as found in the temperature-dependent resistivity ($\rho_{ab}(T)$) in Fig. S10a. Above 15 GPa, $\rho_{ab}(T)$ drops by almost two orders of magnitude, indicating the pressure-driven MIT, while the slope $d\rho_{ab}/dT$ remains positive up to 24 GPa. The resistivity anomaly corresponding to the ferrimagnetic transition temperature T_c also shifts towards higher temperatures, approaching room temperature at $P_c \sim 15.4$ GPa. Above P_c, the anomalous Hall effect becomes significant, similar to the undoped $Mn_3Si_2Te_6$. Using the magnetic susceptibility estimated by the low-field anomalous Hall effect, we tracked T_c at high pressure despite the negligible anomaly in the resistivity $\rho_{ab}(T)$ as shown in Fig. S11b. The resulting T_c as a function of pressure follows a dome-shaped dependence in the pressure-temperature phase diagram (Fig. S10c), resembling those observed in the undoped sample in Fig. 1f of the main text.

Magnetotransport properties of $Mn_3Si_2(Te_{1-x}Se_x)_6$ ($x = 0.2$) up to 24 GPa are presented in Figs. S10c-g. The MR ratio $\rho(0)/\rho(H)$ continuously decreases with increasing pressure and eventually becomes negligible above $P_c \sim 15$ GPa. The Hall conductivity (σ_{xy}) of 20% Se-doped sample is small at low pressures ($P < 15$ GPa), but is suddenly enhanced, reaching a maximum value of ∼16 S cm^{-1} at around 19 GPa. These anomalies in the MR ratio and the Hall conductivity at P_c are similar to the undoped case (Fig. 2). Although Se doping at the Te sites slightly modifies bonding angles and distances, which somewhat reduces T_c as compared to the undoped sample, the pressure-induced magnetic and electronic

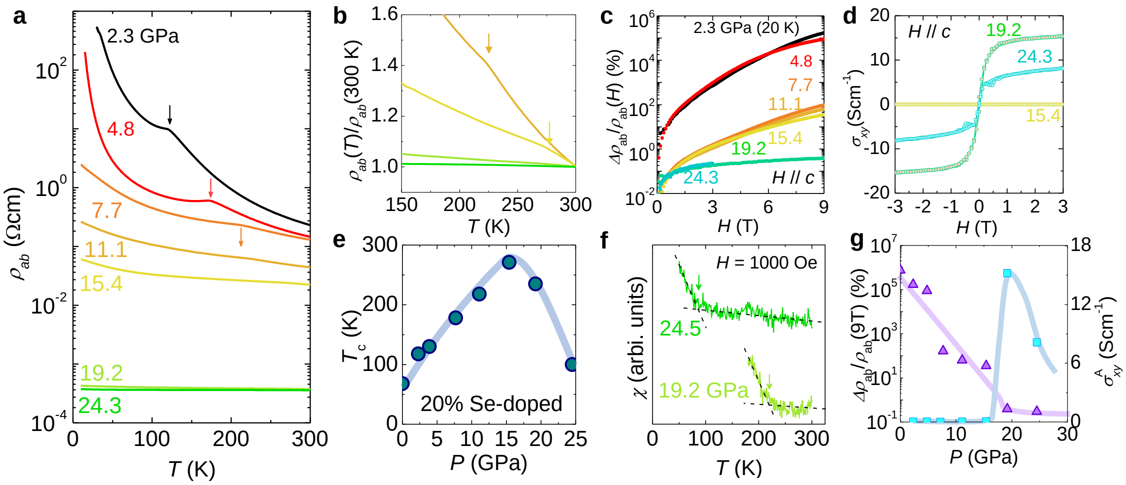

Fig. S10. **Metal-insulator transition and magnetotransport properties of $Mn_3Si_2(Te_{1-x}Se_x)_6$ (x = 0.2) at high pressures.** (a) Temperature-dependent *ab*-plane resistivity $\rho_{ab}(T)$ of $Mn_3Si_2Te_{4.8}Se_{1.2}$ at various pressures. The arrows indicate the resistive anomaly at T_c. (b) The normalized resistivity $\rho_{ab}(T)/\rho_{ab}(300\text{ K})$. (c) the magnetoresistance (MR) ratio ($\Delta\rho/\rho(H)$) and (d) Hall conductivity (σ_{xy}) at 10 K measured at various pressures.(e) Pressure-temperature phase diagram of $Mn_3Si_2Te_{4.8}Se_{1.2}$ showing a dome-shaped behaviour of T_C under pressure. (f) Temperature-dependent magnetic susceptibility $\chi_H(T)$, extracted from $\sigma_{xy}(H)/H$ data above 15 GPa. (g) Pressure-dependence of the MR ratio at 9 T and the anomalous term of Hall conductivity (σ^A_{xy}). The MR ratio is continuously suppressed by pressure and becomes negligible above 15 GPa, where simultaneously σ^A_{xy} becomes significant in the metallic state.

properties, including the concomitant metal-insulator and spin-reorientation transition and a dome-shaped T_c variation, are qualitatively the same. These results suggest that the pressure-driven modulation of the electronic structure plays a critical role in $Mn_3Si_2Te_6$ as discussed in the main text.

Supplementary Note 8. Comparison of pressure-driven transitions in manganese chalcogenides

As discussed in the main text, the pressure-driven phase transition in $Mn_3Si_2Te_6$ has distinct characteristics from those found in other manganese chalcogenides. In Table S2, we compare the features of pressure-driven transitions in $Mn_3Si_2Te_6$ and several Mn-based

Table. S2. **Characteristics of pressure-driven phase transitions in Mn^{2+} chalcogenides.** Types of pressure-driven phase transitions and their critical pressures P_c are listed for Mn^{2+} chalcogenides. The types of magnetic and electronic transitions include spin crossover (SCO), spin reorientation (SRT), charge-transfer-type metal-insulator (CT), Mott-Hubbard type metal-insulator (MH) transitions, characterized by changes in spin and electronic configurations, respectively.

	P_c (GPa)	$\Delta V/V$(%)	Type	Reference
MnS_2	~12	22	SCO, CT	[13, 14]
$MnSe_2$	~ 12.5 – 16.5	19.3	SCO, CT	[15]
$MnTe_2$	~8	~16	SCO, CT	[16]
MnS	30	22.6	SCO, CT	[12]
MnSe	20 – 30	22.0	SCO, CT	[12]
$MnPS_3$	30	19.8	SCO, MH	[11]
$MnPSe_3$	24	20.7	SCO, MH	[11]
$Mn_3Si_2Te_6$	~14	~7	SRT, CT	this work

chalcogenides, including $\mathrm{MnP}Ch_3$[11], $\mathrm{Mn}Ch$[12], and $\mathrm{Mn}Ch_2$ (Ch = S, Se, Te) [13–16]. In other manganese chalcogenides, the metal-insulator transition is associated with a giant volume reduction of $\Delta V/V_0 \sim 20\%$. This strong volume reduction is commonly observed, regardless of the electronic structure type, either Mott-Hubbard or charge-transfer types, which has been attributed to the spin-crossover transition of Mn^{+2} from the high-spin ($t_{2g}^3 e_g^2$) to the low-spin ($t_{2g}^5 e_g^0$) states. In contrast, the metal-insulator transition in $Mn_3Si_2Te_6$ is accompanied by a moderate volume reduction of $\Delta V/V_0 \sim 7\%$, which is three times smaller than the typical values in manganese chalcogenides. The absence of any signature of spin-crossover transition in the field-dependent magnetization measurements, but instead a clear spin-reorientation transition (Fig. 2), corroborates that the metal-insulator transition in $Mn_3Si_2Te_6$ is electronically-driven rather than magnetically-driven.

Supplementary Note 9. Physical properties of $Mn_3Si_2Te_6$ on decompression from high pressure above P_C

We have shown that $Mn_3Si_2Te_6$ undergoes a concomitant MIT, SRT and structural modification under compression above P_c. However, whether this concomitant transition is

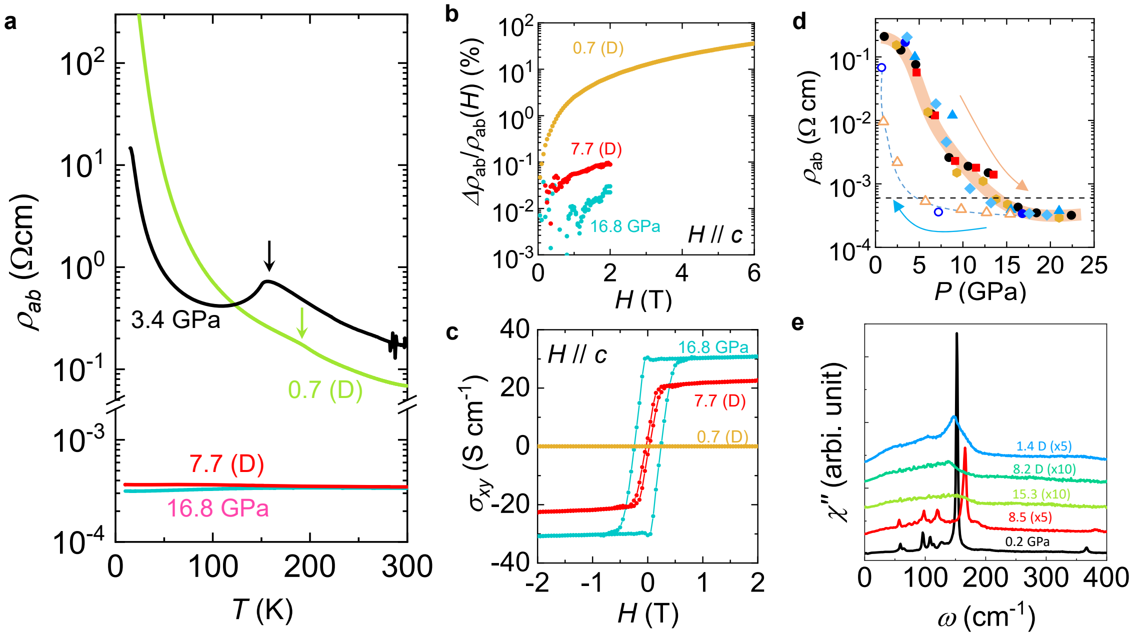

Fig. S11. **Physical properties of $Mn_3Si_2Te_6$ on decompression from high pressure above P_c.** (a) Temperature-dependent ab-plane resistivity $\rho_{ab}(T)$ of $Mn_3Si_2Te_6$ at various pressures during increasing and releasing pressure. Arrows indicate the resistive anomaly at T_c. (b) The magnetoresistance (MR) ratio ($\Delta\rho/\rho(H)$) and (c) Hall conductivity (σ_{xy}) at 15 K measured at various pressures. (d) Pressure-dependent resistivity of $Mn_3Si_2Te_6$ at 300 K showing a significant hysteresis between compression and decompression. (e) Room temperature Raman spectra of $Mn_3Si_2Te_6$ at various pressures. The disappearance of Raman modes on compression above P_C is retained during decompression to ∼1.4 GPa. The label ”D” in all figures represents data taken on decompression.

irreversible and can be decoupled remains unclear. To investigate this issue, we carried out another run where we compressed $Mn_3Si_2Te_6$ to above P_C and subsequently released the pressure. We measured the electronic transport properties at two different pressures of 3.4 GPa ($< P_c$) and 16.8 GPa ($> P_c$) during compression and two additional pressures of 7.7 GPa and 0.7 GPa (close to ambient) during decompression, as presented in Fig. S11a. Consistent with the results in the main text, the insulating behaviour observed at 3.4 GPa ($< P_c$) is suppressed with pressure showing metallic behaviour in the resistivity at 16.8 GPa with a positive slope of $d\rho_{ab}/dT$, confirming the MIT of $Mn_3Si_2Te_6$ above P_c. On releasing pressure to 7.7 GPa, the magnitude of resistivity remains relatively unchanged and $Mn_3Si_2Te_6$ stays in the metallic state. In contrast, the resistivity increases sharply by more

than two orders of magnitude, accompanied by strong insulating behaviour as the pressure is reduced further to below 1 GPa. This behaviour indicates that the band gap re-opens and the semiconducting state of $Mn_3Si_2Te_6$ is recovered upon full decompression.

Magnetotransport properties of $Mn_3Si_2Te_6$ measured on releasing pressure from 16.8 GPa are presented in Figs. S11b-c. The magentoresistance (MR) ratio $\Delta\rho/\rho(H)$, which is negligible in the metallic state above P_c, gradually enhances with decreasing pressure and becomes pronounced in the insulating state at 0.7 GPa. Simutaneously, the large Hall conductivity ($\sigma_{xy}(H)$) in the metallic state is weakened and becomes negligible below 1 GPa. A negligible $\sigma_{xy}(H)$ in the insulating state suggests that the spin direction is no longer along the c-axis and return to the ab-plane upon decompression. These observations are entirely opposite to the behaviour during compression and confirms the reversibility of $Mn_3Si_2Te_6$, albeit with a large hysteresis. Consistently, the pressure-dependent room temperature resistivity exhibits the reversible behaviour with a hysteresis as shown in Fig. S11d.

A significant hysteresis between compression and decompression is also evident from Raman spectroscopy measurements, where the initial Raman modes observed below P_c are not fully recoverable during decompression (Fig. S11e). The room temperature Raman spectrum taken at 1.4 GPa after decompression only shows broad features, common in decompressed samples, and can be explained by residual stress that is not fully relieved within the sample. These results firmly establish that SRT is intimately connected to MIT in $Mn_3Si_2Te_6$. Even with significant strain disorder in the decompressed sample, leading to variation of bond angles and distances between the neighboring Mn atoms that deviate significantly from the ideal values, the preferred spin orientation, either along the in-plane or the out-of-plane directions, is well defined and correlated to the electronic states, either gapped or gapless. Therefore, it can be concluded that indeed MIT and SRT are tied together, and crystal structure modification at P_c could be an additional consequence of the coupled MIT and SRT.

Supplementary Note 10. Infrared spectroscopy at high pressures

Infrared (IR) measurements were conducted using a Bruker vertex 70v interferometer equipped with a Globar MIR source and a KBr beamsplitter. The reflected IR radiation was capture using a x15 (0.4 NA) Cassegrain objective and a LN-MCT D313 detector. All acquisitions were collected in 300 scans and over a frequency range of 400-15000 cm^{-1} with

a resolution of 2 cm^{-1}. The reflectivity spectra were taken from the area of a 50×50 μm^2 at the center of the sample.

Figure S12a shows the IR reflectance spectra of $Mn_3Si_2Te_6$ from 600 to 5000 cm^{-1} obtained at various pressures up to 18 GPa at room temperature. While a Drude-like mode appears in the low-frequency region of all spectra, recent findings indicate that this rise in the reflectance results from a phononic contribution occurring in the far-IR region below 600 cm^{-1} [17]. The reflectivity of $Mn_3Si_2Te_6$ gradually increases with pressure, followed by a sharp rise above ∼13 GPa, similar to the transport results. To get more insights, we fitted all reflectance spectra using a Drude-Lorentz model employed in the RefFIT software [18]. The spectra can be well fitted with at least four Lorentz oscillators for $P < 11$ GPa, while an additional Drude mode was required to fit the spectra for $P > 11$ GPa. The corresponding optical conductivity $\sigma(\omega)$ derived with a standard Kramers-Kronig transformation of the

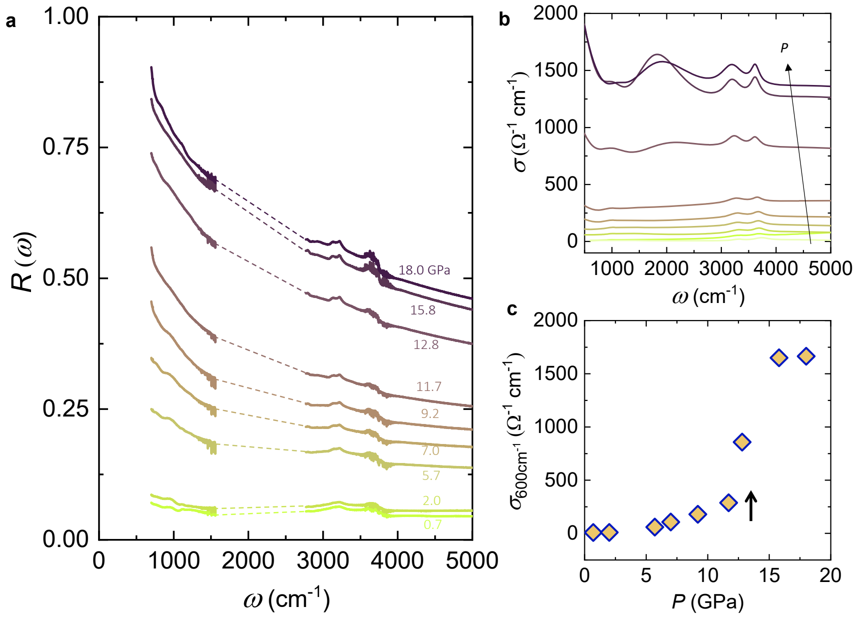

Fig. S12. **Infrared spectroscopy studies of $Mn_3Si_2Te_6$ at high pressure** (a) Room-temperature reflectance spectra of $Mn_3Si_2Te_6$ at various pressures. The data between 1600 cm^{-1} and 2700 cm^{-1} are excluded due to strong diamond absorption (indicated by dashed lines). (b) Real part of the complex optical conductivity $\sigma(\omega)$ at various pressures derived from the fitting to the reflectance spectra. (c) Pressure-dependent optical conductivity at $\omega = 600$ cm^{-1}. A four-fold enhancement of the conductivity above ∼13 GPa is indicated by the arrow.

fitted reflectance spectra are shown in Fig S12b. The optical conductivity $\sigma(\omega)$ increases gradually with pressure up to 11 GPa, above which it enhances anomalously at low energies. Similar behaviour was also observed in VO_2 and attributed to the onset of pressure-induced MIT [19]. The pressure-dependent $\sigma(\omega = 600 \text{ cm}^{-1})$, presented in Fig S12c, exhibits a four-fold enhancement in $\sigma(\omega = 600 \text{ cm}^{-1})$ above $\sim$13 GPa, consistent with the transport results. This optical conductivity enhancement near P_c provides spectroscopic evidence of the MIT at P_c in $Mn_3Si_2Te_6$.

Supplementary Note 11. Comparison with the recent results on $Mn_3Si_2Te_6$ at high pressures

Recent high pressure study on $Mn_3Si_2Te_6$ by Wang *et al.* [20] claimed that the MIT occurs at 1.5-2.5 GPa, much lower than $P_c \sim 14$ GPa in this work. We employed a standard criterion for the critical pressure P_c, above which the system is inferred to be metallic when the temperature-dependent slope of resistivity $d\rho/dT$ shows a positive temperature dependence ($d\rho/dT > 0$). We found excellent agreement between the data from six different crystals from three different batches. The pressure dependent data of the room temperature resistivity collapses into a single curve as shown in Fig. S13a. The crossover between insulating and metallic behaviors is clearly separated by the Mott-Ioffe-Regel (MIR) limit *i.e.* $\rho_{\text{MIR}} = \bar{h}c/e^2$ (the dashed line in Fig. S13a) where c is the c-axis lattice constant of $Mn_3Si_2Te_6$. In addition, the pressure-dependent resistivity estimated from the optical conductivity (Fig. S12c) at low frequency, $\sigma(\omega = 600 \text{ cm}^{-1})$, is consistent with the transport results. Furthermore, the recent study by Huang *et al.* [21] claimed that MIT occurs above 10 GPa which agrees well with our work. As shown in Fig. S13b, the pressure-dependence of normalized resistance $\rho_{ab}(P)/\rho_{ab}(\sim 1 \text{ GPa})$ by Huang *et al.* [21] follows the similar trend to our results albeit exhibiting a slightly different behavior at low pressures. In contrast, the data in Ref. [20] exhibit a rapid decrease at much lower pressure.

As commonly observed in conventional semiconductors, a small amount of vacancies or impurities in $Mn_3Si_2Te_6$ crystals, incorporated during the synthesis, introduce the impurity (accepter) levels near the valence bands, providing charge carriers, as extensively discussed in Ref. [1]. The temperature-dependent and magnetic-field-dependent resistivity of $Mn_3Si_2Te_6$ single crystals can be highly sensitive to the impurity concentration within the crystal. The smaller resistivity and smaller magnetoconductivity indicate higher doping due to a larger

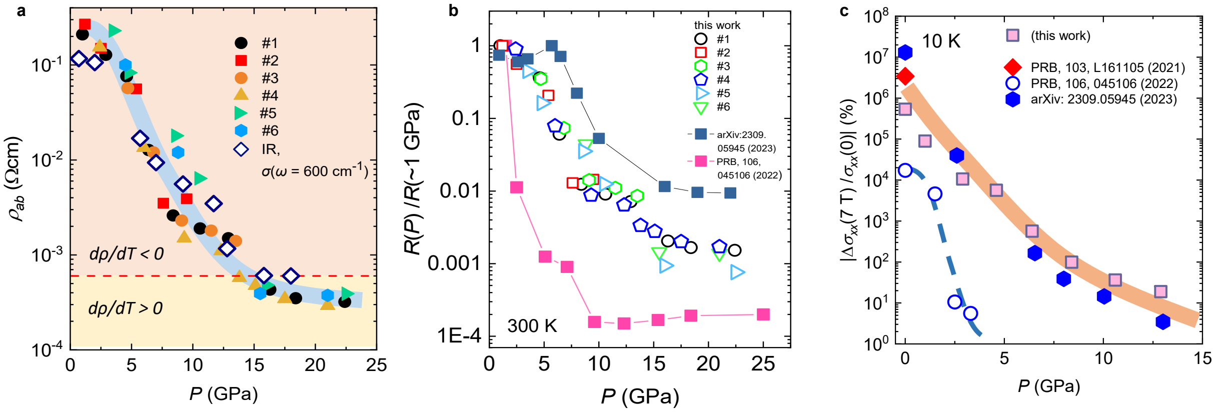

Fig. S13. **Comparison of high pressure transport data on several $Mn_3Si_2Te_6$ crystals.**(a) Pressure dependence of room temperature in-plane resistivity of $Mn_3Si_2Te_6$ crystals studied in this work. For comparison, we included the resistivity value estimated from the optical conductivity at low frequency, $\sigma(\omega = 600\ \text{cm}^{-1})$, which is consistent with the transport data. Dashed line represents the estimated Mott-Ioffe-Regel limit. (b) Comparison of pressure dependence normalized room temperature in-plane resistivity of $Mn_3Si_2Te_6$. (c) Pressure dependence of magnetoconductivity ratio $\Delta\sigma(7\ \text{T})/\sigma(0)$ at 10 K and 7 T of several $Mn_3Si_2Te_6$ crystals reported so far.

amount of impurities. In Fig. S13c, we compare the values of magnetoconductivity (MC) ratio at 10 K and 7 T for several $Mn_3Si_2Te_6$ crystals reported so far [1, 2, 20, 21]. Typically, the MC ratio at 10 K and 7 T, reflecting the MIT induced by the out-of-plane magnetic field is $\sim 10^6\%$ or higher. However the $Mn_3Si_2Te_6$ crystal studied by Wang *et al.* [20] exhibits the smallest MC ratio of $\sim 10^4\%$, at least two orders of magnitude lower than those reported previously. The pressure dependent MC ratio of $Mn_3Si_2Te_6$ crystal grown by Wang *et al.* also differs drastically as compared to those in this work and in Ref. [21], which agree well with each other. The small MC and its rapid reduction under pressure suggest that the $Mn_3Si_2Te_6$ crystal in Ref. [20] is in the high doping regime with a large impurity concentration, which may explain the distinct results in Ref. [20] from those in this work and in Ref. [21].

[1] J. Seo, C. De, H. Ha, J. E. Lee, S. Park, J. Park, Y. Skourski, E. S. Choi, B. Kim, G. Y. Cho, H. W. Yeom, S.-W. Cheong, J. H. Kim, B.-J. Yang, K. Kim, and J. S. Kim, Nature **599**, 576 (2021).

[2] Y. Ni, H. Zhao, Y. Zhang, B. Hu, I. Kimchi, and G. Cao, Phys. Rev. B **103**, L161105 (2021).

[3] H. K. Mao, J. Xu, and P. M. Bell, J. Geophys. Res. **91**, 4673–4676 (1986).

[4] S. Djurdji ć Mijin, A. Šolajić, J. Pešić, Y. Liu, C. Petrovic, M. Bockstedte, A. Bonanni, Z. V. Popović, and N. Lazarević, Phys. Rev. B **107**, 054309 (2023).

[5] D. Louër and A. Boultif, Powder Diffr. **29**, S7–S12 (2014).

[6] V. Favre-Nicolin and R. Černý, J. Appl. Crystallogr. **35**, 734 (2002).

[7] M. Kertesz and R. Hoffmann, J. Am. Chem. Soc. **106**, 3453 (1984).

[8] M. I. Aroyo, J. M. Perez-Mato, C. Capillas, E. Kroumova, S. Ivantchev, G. Madariaga, A. Kirov, and H. Wondratschek, Z. Krist. **221**, 15 (2006).

[9] M. I. Aroyo, A. Kirov, C. Capillas, J. M. Perez-Mato, and H. Wondratschek, Acta Cryst. **A62**, 115 (2006).

[10] A. F. May, H. Cao, and S. Calder, J. Magn. Magn. Mater. **511**, 166936 (2020).

[11] Y. Wang, Z. Zhou, T. Wen, Y. Zhou, N. Li, F. Han, Y. Xiao, P. Chow, J. Sun, M. Pravica, A. L. Cornelius, W. Yang, and Y. Zhao, J. Am. Chem. Soc. **138**, 15751 (2016).

[12] Y. Wang, L. Bai, T. Wen, L. Yang, H. Gou, Y. Xiao, P. Chow, M. Pravica, W. Yang, and Y. Zhao, Angew. Chem. Int. Ed. **55**, 10350 (2016).

[13] S. A. J. Kimber, A. Salamat, S. R. Evans, H. O. Jeschke, K. Muthukumar, M. Tomić, F. Salvat-Pujol, R. Valentí, M. V. Kaisheva, I. Zizak, and T. Chatterji, Proc. Natl. Acad. Sci. USA **111**, 5106 (2014).

[14] D. Durkee, N. Dasenbrock-Gammon, G. A. Smith, E. Snider, D. Smith, C. Childs, S. A. Kimber, K. V. Lawler, R. P. Dias, and A. Salamat, Phys. Rev. Lett. **127** (2021).

[15] B. Wang, X. Wang, S. Wang, D. Tan, W. Xiao, W. Liang, and M. Song, Phys. Chem. Miner. **47**, 41 (2020).

[16] P. Vulliet, J. P. Sanchez, D. Braithwaite, M. Amanowicz, and B. Malaman, Phys. Rev. B **63**, 184403 (2001).

[17] Q. Wu, Q. Yin, S. Zhang, T. Hu, D. Wu, L. Yue, B. Li, S. Xu, R. Li, Q. Liu, H. Lei, T. Dong,

and N. Wang, Pump-induced terahertz conductivity response and peculiar bound state in $mn_3si_2te_6$ (2023), arXiv:2311.14673v1 [cond-mat.mtrl-sci].

[18] A. B. Kuzmenko, Rev. Sci. Instrum. **76**, 083108 (2005).

[19] E. Arcangeletti, L. Baldassarre, D. Di Castro, S. Lupi, L. Malavasi, C. Marini, A. Perucchi, and P. Postorino, Phys. Rev. Lett. **98**, 196406 (2007).

[20] J. Wang, S. Wang, X. He, Y. Zhou, C. An, M. Zhang, Y. Zhou, Y. Han, X. Chen, J. Zhou, and Z. Yang, Phys. Rev. B **106**, 045106 (2022).

[21] C. Huang, M. Huo, X. Huang, H. Liu, L. Li, Z. Zhang, Z. Chen, Y. Han, L. Chen, F. Liang, H. Dong, B. Shen, H. Sun, and M. Wang, Gap and magnetic engineering via doping and pressure in tuning the colossal magnetoresistance in $(mn_{1-x}mg_x)_3si_2te_6$ (2023), arXiv:2309.05945 [cond-mat.mtrl-sci].
